# Validation of a Ramp Running Protocol for Determination of the True VO_2max_ in Mice

**DOI:** 10.3389/fphys.2016.00372

**Published:** 2016-08-29

**Authors:** Mohamed Ayachi, Romain Niel, Iman Momken, Véronique L. Billat, Laurence Mille-Hamard

**Affiliations:** Unité de Biologie Intégrative des Adaptations à l'Exercice, Université d'Evry Val d'EssonneEvry, France

**Keywords:** VO_2max_, mice, exercise protocol, comparative physiology, performance

## Abstract

In the field of comparative physiology, it remains to be established whether the concept of VO_2max_ is valid in the mouse and, if so, how this value can be accurately determined. In humans, VO_2max_ is generally considered to correspond to the plateau observed when VO_2_ no longer rises with an increase in workload. In contrast, the concept of VO_2peak_ tends to be used in murine studies. The objectives of the present study were to determine whether (i) a continuous ramp protocol yielded a higher VO_2peak_ than a stepwise, incremental protocol, and (ii) the VO_2peak_ measured in the ramp protocol corresponded to VO_2max_. The three protocols (based on intensity-controlled treadmill running until exhaustion with eight female FVB/N mice) were performed in random order: (a) an incremental protocol that begins at 10 m.min^−1^ speed and increases by 3 m.min^−1^ every 3 min. (b) a ramp protocol with slow acceleration (3 m.min^−2^), and (c) a ramp protocol with fast acceleration (12 m.min^−2^). Each protocol was performed with two slopes (0 and 25°). Hence, each mouse performed six exercise tests. We found that the value of VO_2peak_ was protocol-dependent (*p* < 0.05) and was highest (59.0 ml.kg ^0.75^.min^−1^) for the 3 m.min^−2^ 0° ramp protocol. In the latter, the presence of a VO_2max_ plateau was associated with the fulfillment of two secondary criteria (a blood lactate concentration >8 mmol.l^−1^ and a respiratory exchange ratio >1). The total duration of the 3 m.min^−2^ 0° ramp protocol was shorter than that of the incremental protocol. Taken as a whole, our results suggest that VO_2max_ in the mouse is best determined by applying a ramp exercise protocol with slow acceleration and no treadmill slope.

## Introduction

Although rodents are often used as models in exercise physiology, there is no consensus on the use of a standardized exercise protocol for determining the maximum oxygen uptake (VO_2max_) in these species. In fact, the concept of peak oxygen consumption (VO_2peak_) is preferred in mice. Given that VO_2max_ is the main determinant of performance in human exercise physiology (i.e., the greatest possible oxygen uptake during physical exercise involving a large proportion of the total muscle mass (Cohn, 1987), it remains to be established whether this concept is valid in the mouse and, if so, how VO_2max_ can be accurately determined.

It is widely acknowledged that VO_2max_ in humans corresponds to both the cardiovascular system's functional limitation and the organism's aerobic capacity. Since the VO_2max_ concept was introduced by Hill and Lupton (1923), the use of exercise protocols with progressive or stepped increments has been validated in human - although the optimal choice of exercise protocol is still subject to debate. In stepwise protocols, the height of the step (i.e., the magnitude of the increment) and the duration of each workload level are left to the investigator's discretion.Since the 1960s, a number of different incremental protocols (with variations in running speed, treadmill slope or both) have been tested for their reliability in determining VO_2max_ (Balke and Ware, 1959; Bruce et al., 1963; Froelicher et al., 1974). In contrast, ramp protocol are characterized by a continuous, gradual increase in the workload (i.e., power, speed or slope) up to maximum values. Many researchers have compared incremental protocols with ramp protocols, in order to establish the most efficient method for determining VO_2max_ (Whipp et al., 1981; Astorino et al., 2005; Yoon et al., 2007). These studies have shown that the ramp exercise protocol is well suited to the human's aerobic metabolism and thus enables VO_2max_ to be accurately determined. However, ramp protocols take longer to complete, and incremental protocols are preferred for the routine measurement of VO_2max_ because they allow other performance indicators (such as the ventilatory threshold and the lactate threshold) to be determined. In humans, VO_2max_ is generally considered to correspond to the plateau observed when VO_2_ no longer increases with speed. However, about half of tested subjects do not reach a plateau before they abandon the protocol; secondary criteria then have to be used to establish when the last (peak) VO_2_ value indeed corresponds to VO_2max_. Three secondary criteria have been proposed: (i) the maximum heart rate at the end of the test, which corresponds to an estimate of the theoretical maximum (Åstrand, 1952; Astrand, 1960; Maritz et al., 1961); (ii) an end-of-exercise respiratory exchange ratio (RER) >1.15 (Issekutz et al., 1962); and (iii) an end-of-exercise blood lactate concentration >8 mmol.l^−1^.

For the purposes of comparative physiology, VO_2max_ has also been determined in rodents. This parameter can be used in studies of exercise training or in descriptive studies of genetically modified animals (Kemi et al., 2002; Hoydal et al., 2007; Mouisel et al., 2014). As in humans, the relationship between running intensity and oxygen uptake is linear in mice (as demonstrated during steady-state, fixed-intensity running (Fernando et al., 1993; Schefer and Talan, 1996; Wisløff et al., 2001); this enables the use of incremental protocols. However, various strains of mouse have been used, and an effect of strain on treadmill performance has been evidenced. FVB mice achieve high maximum and critical speeds during forced treadmill exercise (Lightfoot et al., 2001; Lerman et al., 2002; Billat et al., 2005). Furthermore, age (Schefer and Talan, 1996) gender (Hoydal et al., 2007) may affect VO_2max_. VO_2peak_ decreases in old age, although female and male mice appear to have similar levels of performance (Kemi et al., 2002; Billat et al., 2005). Consequently, the disparities in the literature data on VO_2peak_ can be explained (at least in part) by differences in age and strain.

Although, the mouse has been widely used to study the biochemical and molecular adaptations to exercise, a number of different protocols have been applied; this may explain (at least in part) the broad range of values obtained for VO_2peak_. Furthermore, it has been reported that VO_2peak_ in mice is slope-dependent (Kemi et al., 2002). The incremental protocols described in the literature differ in their duration, increment size and the criteria used to determine exhaustion (usually the animal's behavior or the shape of the VO_2_/time curve) (Dohm et al., 1994; Rezende et al., 2005; Hawkins et al., 2007). It is not known whether a ramp protocol is suitable for determining VO_2peak_ in mice or whether this value is protocol-dependent. Kemi et al. (2002) were the first to estimate the animal's level of exhaustion by applying secondary criteria (i.e., the RER and blood lactate levels) (Kemi et al., 2002). However, the presence or absence of a VO_2_ plateau, the latter's characteristics and the relationship between VO_2peak_ and VO_2max_ have not previously been studied in the mouse. We hypothesized that VO_2peak_ and VO_2max_ in mice are protocol-dependent and that (as in humans) a ramp exercise protocol would be suitable for determining VO_2max_. Thus, the objective of the present study in mice was to determine whether (i) a continuous ramp protocol yielded a higher VO_2peak_ than a stepwise, incremental protocol, and (ii) the VO_2peak_ measured in the ramp protocol corresponded to VO_2max._

## Methods

### Animal

One-year-old male FVB mice (*n* = 8) were selected for use in this study by virtue of their high level of performance on a treadmill (Lerman et al., 2002). The mice were kept in a specific and opportunistic pathogen-free animal facility (CERFE, Genopole, Evry, France) at a temperature of 22°C and with light-dark cycles 12/12-h. The animals were fed a standard diet *ad libitum*. Our protocol was approved by our institutions Animal Care and Use Committee on Care and complied with the European Convention of the Council of Europe for the protection of vertebrate animals used for experimental and other scientific purposes.

### Familiarization

Mice were familiarized with the single-lane, motorized treadmill (adjustable belt speed: 0–99.9 m.min^−1^; Columbus Instruments, Columbus, OH, USA) during four 10-min running sessions (at 0, 3, 6, and 9 m.min^−1^), with a 48-h interval between each session. All mice subsequently included in the study were able to run for the required time at 9 m.min^−1^. The running speed was not increased further, in order to avoid a training effect.

### The exercise protocol

The treadmill was set up in a metabolic chamber. Three different protocols were applied: an incremental protocol (IP) with a starting speed of 10 m.min^−1^ and an increment of 3 m.min^−1^ every 3 min; a ramp protocol with a starting speed of 3 m.min^−1^ and an acceleration of 0.05 m.min^−1^.s^−1^ (corresponding to 3 m.min^−2^), hereafter referred to as “Ramp3”; and a ramp protocol with a starting speed of 3 m.min^−1^ and an acceleration of 0.2 m.min^−1^.s^−1^ (corresponding to 12 m.min^−2^), hereafter referred to as “Ramp12.” Each of the three protocols was performed with two different slopes (0 and 25°); hence, each mouse performed six sessions. To avoid conditioning bias, the test sequence was randomized and there was 24-h interval between each session. The exercise session lasted until exhaustion, which was defined as the mouse's inability to maintain running speed despite being in contact with the electrical grid for more than 5 consecutive seconds (Mille-Hamard et al., 2012). All mice were compliant in all tests. The resting blood lactate concentration was measured at the start of the test ([Lac]_rest_) and 2 min after the end of each run ([La]_max_). To this end, a blood drop was collected at the tail vein (using the tail snip method), placed on a test strip and inserted into a lactate analyzer (Lactate Pro, Arkray, Inc., Kyoto, Japan).

### Gas measurements

Ambient air was fed through the metabolic chamber at a rate of 0.66 l.min^−1^; the flow was chosen such that the incoming vs. outgoing difference in O_2_ fraction was within the sensor's range of measurement (−0.3 to −0.8% O_2_). A fan was used to mix the incoming air with the air around the treadmill and blow it toward the animal. The air flowed from the front of the treadmill to the rear of the treadmill and then returned toward the front under the belt. This created a rapid, circular “loop” of mixed gases (i.e., incoming “fresh” air mixed with the accumulated, exhaled gases), from which a sample was drawn for analysis every 5 s. Samples were dried prior to measurement of the O_2_ and CO_2_ fractions. The gas analyzers were calibrated with standardized gas mixtures (Air Liquide Santé, Paris, France) before each test session, as recommended by the manufacturer. To allow rapid comparisons over a wide range of body weights (especially with human data), dimensional analyses and empirical studies have shown that VO_2_ should be divided by the body mass raised to the power of 0.75 (Taylor et al., 1981; Hoydal et al., 2007; Mille-Hamard et al., 2012).

### Data analysis

VO_2peak_ was defined as the highest observed value of VO_2_ when averaged over successive 15 s periods. VO_2max_ was defined as in humans (i.e., the highest VO_2peak_ value recorded during a set of different test protocols, and the occurrence of a VO_2_ plateau). The VO_2_ plateau was determined when the VO_2_ did not increase by more than 1% of the difference between the VO_2_ at rest and VO_2peak_ over a 30 s period, despite an increase in running speed. The mouse's maximum speed (V_max_) was defined as the running speed at the end of the protocol. The RER was defined as the ratio between the amount of oxygen (O_2_) consumed and the amount of carbon dioxide (CO_2_) produced in the metabolic chamber. The maximum respiratory exchange ratio (RER_max_) was defined as the highest observed value of the RER when averaged over successive 15 s periods.

### Statistics

Data are expressed as the mean ± standard deviation (SD). Statistical analysis was carried out with a two-way repeated measures ANOVA, followed by a Holm-Sidak *post-hoc* test. The threshold for statistical significance was set to *p* < 0.05. All statistical analyses were performed using STATISTICA software (version 9.0, Statsoft, Berkeley, CA, USA).

## Results

### VO_2peak_ in each exercise protocol

**Figure 1:**
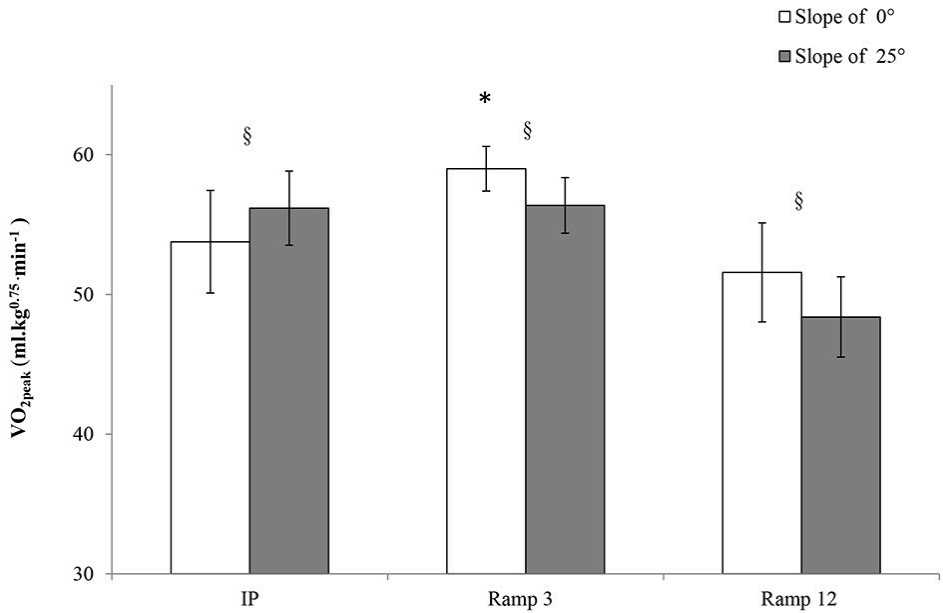
**VO_2peak_ in each exercise protocol** : one-year-old sedentary FVB/N mice (*n* = 8) performed six exhaustive exercise protocols with a treadmill slope of 25 or 0°. IP, an incremental protocol with a starting speed of 10 m.min^−1^ and an increment of 3 m.min^−1^ every 3 min; Ramp3, a ramp protocol with a starting speed of 3 m.min^−1^ and an acceleration of 3 m.min^−2^ (0.05 m.min^−1^.s^−1^); Ramp12, a ramp protocol with a starting speed of 3 m.min^−1^ and an acceleration of 12 m.min^−2^ (0.2 m.min^−1^.s^−1^); §, a significant difference between 25 and 0° for the same protocol (*p* < 0.05); ^*^, differs significantly from all other protocols (*p* < 0.05).

The highest observed VO_2peak_ (59.0 ± 0.61 ml.kg^−0.75.^min^−1^, Figure 1) was obtained during the Ramp3 0° protocol. This value was significantly greater than those obtained in the other protocols. The presence of a slope influenced the value of VO_2peak_, which was higher in IP 25° than in IP 0° but lower in Ramp3 25° and Ramp12 25° than in Ramp3 0° and Ramp12 0°. The minimum VO_2_ determined at the beginning of the protocol (referred to as the VO_2_ at rest) was essentially the same in all protocols (mean: 43.6 ± 3.9 ml.kg^−0.75.^min^−1^).

### Observation of a VO_2peak_ plateau as a function of the exercise protocol

As shown in Table 1, all mice displayed a VO_2_ plateau for at least 30 s during the Ramp3 0° and IP 25° protocols (mean plateau duration: 57.5 s ± 11.3 and 75 ± 11.24 s, respectively). During other protocols, some (but not all) mice reached a VO_2_ plateau for at least 30 s (Table 1)

**Table 1:** **Percentages of mice reaching a VO_2_ plateau for least 30 s, as defined in the Methods section**.

	**IP (%)**	**Ramp3 (%)**	**Ramp12 (%)**
Slope of 0°	87.5	100	87.5
Slope of 25°	100	75	87.5

*IP, an incremental protocol with a starting speed of 10 m.min^−1^ and an increment of 3 m.min^−1^ every 3 min; Ramp3, a ramp protocol with a starting speed of 3 m.min^−1^ and an acceleration of 3 m.min^−2^ (0.05 m.min^−1^.s^−1^); Ramp12, a ramp protocol with a starting speed of 3 m.min^−1^ and an acceleration of 12 m.min^−2^ (0.2 m.min^−1^.s^−1^)*.

### Maximal respiratory exchange ratio: RER_max_

**Figure 2:**
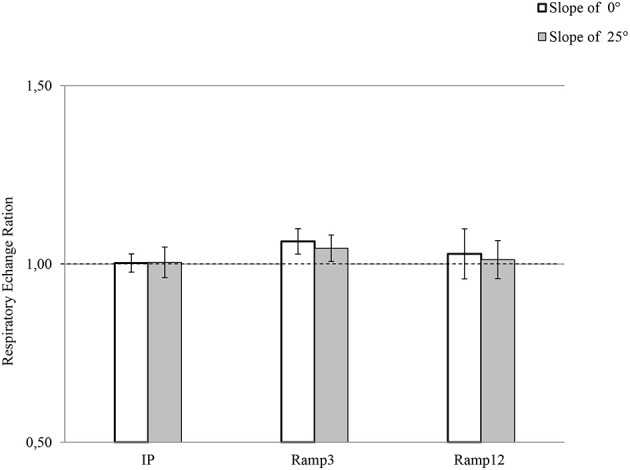
**RER_max_ in each exercise protocol**. IP, an incremental protocol with a starting speed of 10 m.min^−1^ and an increment of 3 m.min^−1^ every 3 min; Ramp3, a ramp protocol with a starting speed of 3 m.min^−1^ and an acceleration of 3 m.min^−2^ (0.05 m.min^−1^.s^−1^); Ramp12, a ramp protocol with a starting speed of 3 m.min^−1^ and an acceleration of 12 m.min^−2^ (0.2 m.min^−1^.s^−1^).

There were no inter-test differences in RER_max_ (Figure 2). For Ramp3 0°, the mean RER_max_ value was 1.06 ± 0.01, and RER_max_ was greater than 1.05 for seven of the eight mice.

### Maximum blood lactate concentration

**Figure 3:**
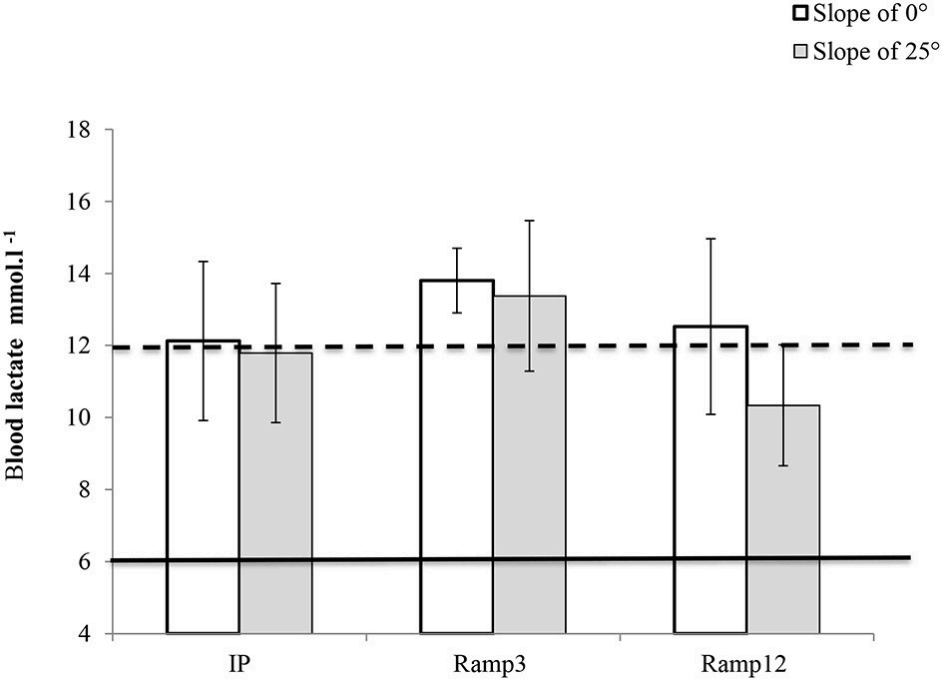
**[La]_max_ (measured 2 min after the end of the run) in each exercise protocol**. IP, an incremental protocol with a starting speed of 10 m.min^−1^ and an increment of 3 m.min^−1^ every 3 min; Ramp3, a ramp protocol with a starting speed of 3 m.min^−1^ and an acceleration of 3 m.min^−2^ (0.05 m.min^−1^.s^−1^); Ramp12, a ramp protocol with a starting speed of 3 m.min^−1^ and an acceleration of 12 m.min^−2^ (0.2 m.min^−1^.s^−1^).

[La]_max_ was above 6 mmol.l^−1^ for all mice and all protocols (Figure 3). In the Ramp3 0° protocol, the mean [La]_max_ was 13.80 ± 0.34 and [La]_max_ was greater than 12 mol.l^−1^ for all mice.

### Maximal speed: V_max_

**Figure 4:**
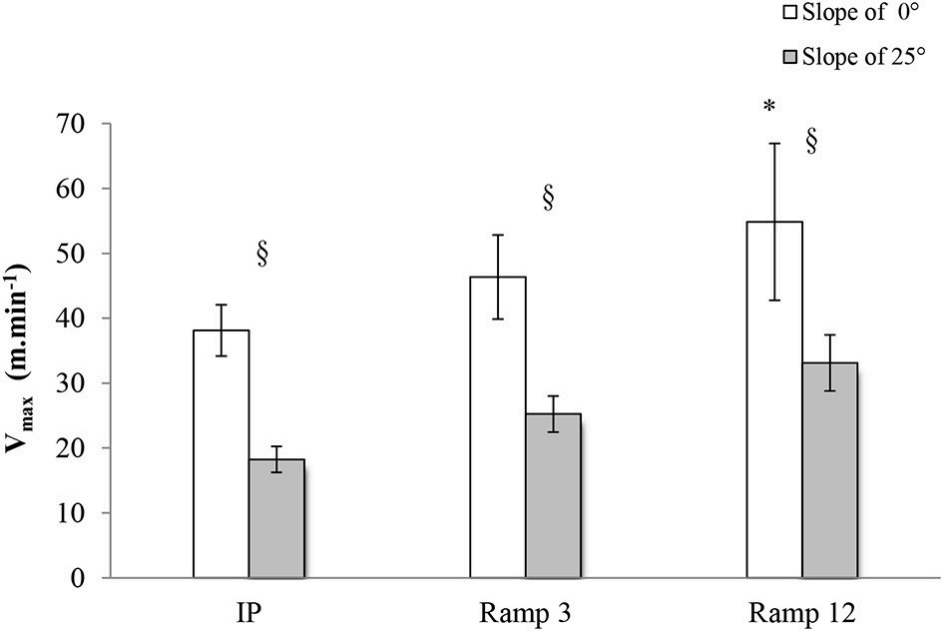
**V_max_ in each exercise protocol**. IP, an incremental protocol with a starting speed of 10 m.min^−1^ and an increment of 3 m.min^−1^ every 3 min; Ramp3, a ramp protocol with a starting speed of 3 m.min^−1^ and an acceleration of 3 m.min^−2^ (0.05 m.min^−1^.s^−1^); Ramp12, a ramp protocol with a starting speed of 3 m.min^−1^ and an acceleration of 12 m.min^−2^ (0.2 m.min^−1^.s^−1^); §, a significant difference between 25 and 0° for the same protocol (*p* < 0.05); ^*^, differs significantly from all other protocols (*p* < 0.05).

The V_max_ of the mice was higher in the ramp protocols (54.88 ± 4.57 m.min^−1^ for Ramp12 0°; 46.34 ± 2.45 m.min^−1^ for Ramp3 0°) than in the step protocol (IP 0°: 38.13 ± 1.79 m.min^−1^) (Figure 4). For all three protocols, V_max_ was higher with 0° than with 25°.

### Time to exhaustion

**Figure 5:**
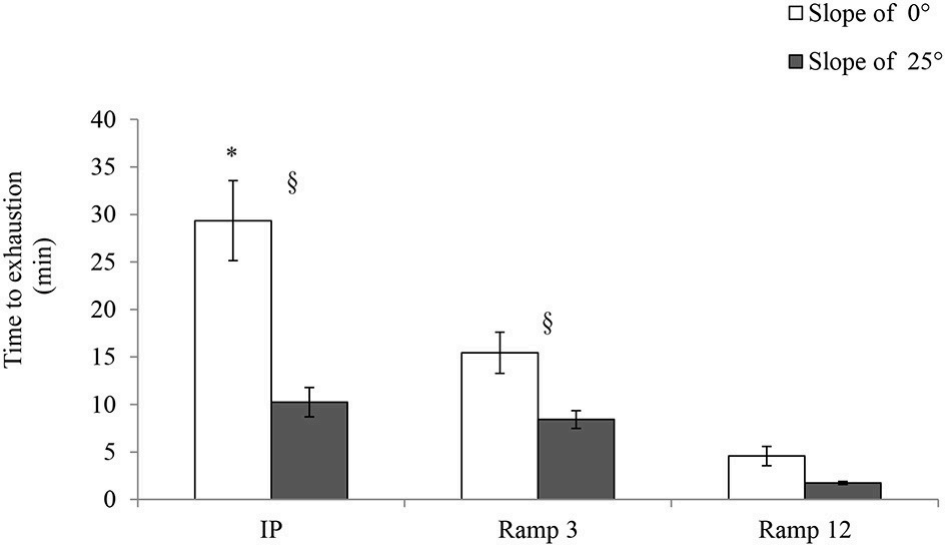
**Time to exhaustion**. IP, an incremental protocol with a starting speed of 10 m.min^−1^ and an increment of 3 m.min^−1^ every 3 min; Ramp3, a ramp protocol with a starting speed of 3 m.min^−1^ and an acceleration of 3 m.min^−2^ (0.05 m.min^−1^.s^−1^); Ramp12, a ramp protocol with a starting speed of 3 m.min^−1^ and an acceleration of 12 m.min^−2^ (0.2 m.min^−1^.s^−1^); §, a significant difference between 25 and 0° for the same protocol (*p* < 0.05); ^*^, differs significantly from all other protocols (*p* < 0.05).

As shown in Figure 5, the time to exhaustion was significantly longer in IP 0° (29.33 ± 1.58 min) than in the two ramp protocols. For example, the time to exhaustion in Ramp3 0° (15.43 ± 0.8 min) was almost half that observed in IP 0°.

## Discussion

The present study in mice was designed to determine whether (i) a continuous ramp protocol yielded a higher VO_2peak_ than a stepwise, incremental protocol, and (ii) the VO_2peak_ measured in the ramp protocol corresponded to VO_2max_.

This is an important issue, given that mice are frequently studied models in exercise physiology and that a variety of exercise protocols have been applied in this context. Our main findings were that a ramp protocol (with an acceleration of 3 m.min^−2^ and no treadmill slope) elicited a higher VO_2peak_ than an incremental protocol (regardless of slope), and that the VO_2peak_ does appear to correspond to the VO_2max_ (given that a VO_2_ plateau was observed and the secondary criteria were met). The Ramp3 0° protocol is therefore relevant for the determination of VO_2max_ inmice.

According to the literature data, VO_2peak_ in sedentary male mice ranges from 47 to 94 ml.kg^−0.75^.min^−1^ (Dohm et al., 1994; Schefer and Talan, 1996; Desai et al., 1999; Niebauer et al., 1999; Kemi et al., 2002). Furthermore, no major gender differences have been reported. Although gender differences have been observed for voluntary exercise (with young female mice running farther and faster than young males Lightfoot et al., 2004; Bartling et al., 2016), studies of forced exercise on a treadmill have not evidenced gender differences for critical speed, maximum distance (Billat et al., 2005; Lightfoot et al., 2007), or VO_2peak_ in untrained mice (Kemi et al., 2002). Hence, we conclude that aerobic capacity does not depend on gender in untrained mice. Along with heterogeneity in the test protocols, several other factors may influence the observed VO_2peak_. It has been reported that VO_2peak_ falls from 79 ml.kg^−0.75^.min^−1^ in young adult (12-month-old) mice to 56 ml.kg^−0.75^.min^−1^ in elderly (24-month-old) mice (Schefer and Talan, 1996). Thus, age differences in various studies may account for some of the discrepancies between reported VO_2peak_ values. Moreover, the mouse's level of performance is known to depend on the strain (Lightfoot et al., 2001; Billat et al., 2005). Given that VO_2peak_ is considered to be an indicator of performance, one can legitimately hypothesize that this variable is also influenced by the strain of mouse studied. The only study to date of VO_2peak_ in FVB mice reported a value corresponding to 60 ml.kg^−0.75^.min^−1^ (Chow et al., 2007) which falls within the range of values observed in the present study. Hence, the choice of different strains may also account for some of the discrepancies in VO_2peak_ values.

Furthermore, the impact of the exercise protocol used to determine VO_2peak_ values in mice has not previously been assessed. To the best of our knowledge, the only previous study in this field focused on the effect of treadmill slope on VO_2peak_ in an incremental protocol (Wisløff et al., 2001). We hypothesized that the choice of exercise protocol would have a critical impact on the measured VO_2peak_. For example, Kemi et al.'s (2002) study used an incremental protocol with an increment of 1.8 m.min^−1^ every 2 min. They reported a mean VO_2peak_ value of 47 ml.kg^−0.75^.min^−1^ and a mean time to exhaustion of 30 min. In contrast, Dohm et al. (1994) study used an incremental protocol with an increment of 8.4 m.min^−1^ every 2 min to obtain a mean VO_2peak_ value of 94 ml.kg^−0.75^.min^−1^ and a time to exhaustion of 16 min. The results of the present study showed that the VO_2peak_ value is protocol-dependent (*p* < 0.05). The highest value was obtained in the Ramp3 0° protocol; hence, ramp protocols are suitable for determining VO_2peak_ in mice. Indeed, the ramp protocol was associated with a shorter time to exhaustion (15 ± 0.82 min in Ramp3 0° and 30 ± 1.51 min in IP 0°). This may explain why VO_2peak_ was higher in the Ramp3 0° protocol than in the IP 0° protocol. In humans, a shorter time to exhaustion is associated with a higher VO_2max_ (Froelicher et al., 1974); this also appears to be true in the mouse.

It has been demonstrated that VO_2peak_ is highest when the treadmill slope is between 15 and 35° (Kemi et al., 2002). Accordingly, we chose a value of 25°. This slope was associated with significant differences in the measured VO_2peak_ (relative to the 0° condition, and for both the incremental protocol and the ramp protocols). Interestingly, the IP 25° protocol yielded a higher VO_2peak_ value that the IP 0° protocol. This confirmed the results of Kemi et al.'s study of an incremental protocol (2002). In contrast, VO_2peak_ was lower for Ramp3 25° than for Ramp3 0°. In exercising human (in whom energy expenditure is mainly related to muscle work), concentric work requires 3- to 5-fold more energy than the same amount of eccentric work. The energy cost of running therefore depends on the relative proportions of these two types of work, which in turn depends on the slope; the steeper the slope at a given speed, the greater the proportion of concentric work and thus the greater the energy expenditure. (Minetti et al., 1993, 1994; Pringle et al., 2002). This phenomenon seems to have occurred in the ramp protocols because the mice attained a lower V_max_ when the treadmill was inclined. Furthermore, running on a sloping treadmill may recruit a greater muscle mass (Kemi et al., 2002). Consequently, involvement of a greater muscle mass and a greater proportion of concentric work in ramp protocols with slope might be responsible for fatigue and thus a lower VO_2peak_. However, the data collected in the present study did not enable us to confirm this hypothesis. Furthermore, it is possible that use of a shallower slope would have increased the concentric work without leading to too much fatigue and thus would have yielded a higher VO_2peak_ value.

As well as being associated with the highest VO_2peak_ value, the Ramp3 0° protocol produced a VO_2max_ plateau for which two secondary criteria (the blood lactate concentration and the RER) were fulfilled. Thus, a ramp protocol with an acceleration of 3 m.min^−2^ and no slope enables the determination of the VO_2max_ in mice, according to the definition usually applied in humans. Over the last 15 years, a number of researchers have evaluated the influence of data sampling on changes over time in VO_2_ and the determination of VO_2max_ in human (Astorino et al., 2005; Midgley et al., 2006, 2007; Astorino, 2009). These studies showed that averaging VO_2_ over successive 15 s periods provided a more accurate measurement of VO_2max_ and increased the likelihood of observing a VO_2_ plateau. As breath-by-breath sampling is not possible for mice in a metabolic chamber, we used the device's shortest sampling time (5 s, i.e., below the maximum recommended value of 15 s). Furthermore, very few studies have focused on whether a VO_2_ plateau (which defines VO_2max_) can be observed in mice. Many researchers have not distinguished between VO_2peak_ and VO_2max_, and have defined VO_2max_ in different ways. For example, Gebczynski defined VO_2max_ as the highest mean VO_2_ value over 1 min (Gebczynski and Konarzewski, 2009), and Ferreira et al. (2007) considered that VO_2max_ was equivalent to VO_2peak_ (Ferreira et al., 2007). In contrast, some researchers have stated that VO_2max_ corresponds to the VO_2_ plateau; unfortunately, the researchers evaluated the VO_2_ curve visually and did not define criteria for detecting a plateau (Niebauer et al., 1999; Kemi et al., 2002). In 1955, Taylor et al. stated that the change in VO_2_ (ΔVO_2_) should be below 2.1 ml.kg^−1^.min^−1^ or 150 ml min^−1^ for more than 30 s if it is to be considered as a VO_2max_ plateau: (Billat et al., 2013). For a sedentary subject, this ΔVO_2_ represents around 5% of the difference between the VO_2_ measured at rest and VO_2max_. In view of our previous data in mice, (Mille-Hamard et al., 2012; Mouisel et al., 2014) and studies indicating that there is not much difference between VO_2_ at rest and VO_2peak_ in mice (Ferreira et al., 2007; Mazzucatto et al., 2014), we decided to reduce the value of ΔVO_2_. Hence, in the present study, the VO_2_ plateau was determined when the VO_2_ did not increase by more than 1% of the difference between the VO_2_ at rest and VO_2peak_ over a 30 s period, despite an increase in running speed.

Furthermore, Kemi et al. considered two of the secondary criteria applied in human exercise tests. Given that non-invasive measurement of the heart rate is not practical in mice, Kemi et al. suggested that an RER > 1 and an [La]_max_ > 6 mmol.l^−1^ can be used to confirm the value of VO_2max_ when a VO_2_ plateau is not observed (Kemi et al., 2002). Our present data on RER_max_ and [La]_max_ suggest that the VO_2max_ was attained by all the mice during the Ramp3 0° protocol. The recorded values of RER_max_ (mean: 1.06 ± 0.01) and [La]_max_ (>12 mmol.l^−1^) indicated that exercise was strenuous. (Astorino, 2009).

In humans, a standardized stepwise protocol is usually preferred because it enables the determination of other performance indices (blood lactate, ventilatory thresholds, heart rate, etc.) as well as VO_2max_. In mice, these indices cannot be calculated without using non-routine equipment (an implanted heart rate sensor and a mouthpiece, for example), and so the ramp protocol suggested here (which enables the true VO_2max_ to be determined rapidly) should be preferred. However, it remains to be seen whether the ramp protocol is suitable for all strains and age groups and for both sexes.

## Conclusion

The principal findings of this study in the mouse were that (i) the VO_2peak_ observed at the end of exhaustive exercise is protocol-dependent, and (ii) a ramp exercise protocol with an acceleration of 3 m.min^−2^ (i.e., 0.05 m.min^−1^.s^−1^) and no treadmill slope is suitable for determining VO_2max_ as defined in humans.

## Author contributions

MA, RN contributed to the design of the work, the acquisition, analysis, and interpretation of data, drafted the work; LM, IM contributed to the design of the work, the acquisition, analysis, and interpretation of data, drafted the work and revisited it critically for important intellectual content; VB contributed to the design of the work, the interpretation of data, revisited the work critically for important intellectual content. All authors approved the version to be published and agreed to be accountable for all aspects of the work in ensuring that questions related to the accuracy or integrity of any part of the work are appropriately investigated and resolved.

### Conflict of interest statement

The authors declare that the research was conducted in the absence of any commercial or financial relationships that could be construed as a potential conflict of interest.
